# A Clinicopathological and Molecular Analysis of Sellar/Suprasellar Neurocytoma Mimicking Pituitary Adenoma

**DOI:** 10.3389/fendo.2022.861540

**Published:** 2022-05-18

**Authors:** Lifeng Zhang, Weiwei Fu, Limei Zheng, Fangling Song, Yupeng Chen, Changzhen Jiang, Zhen Xing, Chengcong Hu, Yuhong Ye, Sheng Zhang, Xiaorong Yan, Xingfu Wang

**Affiliations:** ^1^ Department of Endocrinology, Fujian Provincial Governmental Hospital, Fuzhou, China; ^2^ Department of Pathology, The Affiliated Hospital of Qingdao University, Qingdao, China; ^3^ Department of Pathology, The First Affiliated Hospital of Fujian Medical University, Fuzhou, China; ^4^ Department of Neurosurgery, The First Affiliated Hospital of Fujian Medical University, Fuzhou, China; ^5^ Department of Radiology, The First Affiliated Hospital of Fujian Medical University, Fuzhou, China

**Keywords:** neurocytoma, extraventricular neurocytoma, sellar and suprasellar region, pituitary tumor, clincopathology

## Abstract

**Objective:**

To investigate the clinicopathological characteristics, molecular genetic characteristics and prognosis of extraventricular neurocytoma located in the sellar/suprasellar region.

**Methods:**

Seven archived tumor samples derived from 4 patients with neurocytoma in the sellar/suprasellar region were collected from the First Affiliated Hospital of Fujian Medical University and the Affiliated Hospital of Qingdao University and retrospectively analyzed for clinical manifestations, imaging features, and histopathological features. Neuronal and pituitary biomarkers and molecular features were detected in these tumor tissues by immunohistochemistry and FISH or Sanger sequencing. The related literature was reviewed.

**Results:**

Three patients were female, while 1 was male, with an average age of 35.5 years (range: 27 to 45 years). The initial manifestations were mainly headache and blurred vision in both eyes. The first MRI examination showed marginally enhancing masses in the intrasellar or intra- to suprasellar region. The diagnosis of pituitary adenomas was based on imaging features. The levels of pituitary hormones were normal. Histologically, the tumor cells were arranged in a sheet-like, monotonous architecture and were uniform in size and shape with round to oval, exquisite and hyperchromatic nuclei, which densely packed close to one another and were separated only by a delicate neuropil background. There was no evident mitosis, necrosis or microvascular proliferation. The three cases of recurrent tumors were highly cellular and showed increased mitotic activity. Immunohistochemically, the tumor cells were positive for syn, CR, CgA, and vasopressin and were focally positive for NeuN, TTF-1, NF, CK8, vimentin, and S100 proteins. Other markers, including IDH1, BRAF VE1, Olig-2, and EMA, were negative. Pituitary transcription factors and anterior pituitary hormones were negative. Molecular genetic testing showed that the tumor cells lacked IDH gene mutations, LOH of 1p/19q, MYCN amplification, and EGFR alteration. With a median follow-up of 74.5 months (range 23 to 137 months), 3 patients relapsed at 11, 50, and 118 months after the initial surgery.

**Conclusion:**

The morphological features and immunophenotypes of neurocytoma in the sellar/suprasellar region are similar to those of classic central neurocytoma. The prognosis is relatively good. Gross-subtotal resection and atypical subtype may be related to tumor recurrence.

## Introduction

The first case of neurocytoma was reported by Hassoun et al. in 1982, who described two cases of third ventricle tumors with neuronal differentiation and proposed the term “central neurocytoma” (CN) (1). Thereafter, tumors with clinicopathological features similar to central neurocytoma were found outside the ventricles, including the hemispheric parenchyma, cerebellum, pons, spinal cord, cauda equina, and retina, which have been termed “extraventricular neurocytoma” (EVN) (2). In 2007, EVN was officially recognized by the World Health Organization (WHO) classification of central nervous system tumors as a distinct entity among glioneuronal tumors, making up 10% of all neurocytomas.

The radiological, histopathological, and immunophenotypic features of EVNs resemble those of CNs. Contrary to CNs, EVNs have a marked tendency for ganglionic differentiation. In addition, EVNs have molecular characteristics distinct from those of CNs (3). The prognosis is generally good for patients with CNs or EVNs who can undergo complete removal. EVN arising in the sellar or suprasellar region is an extremely rare tumor proposed to be within the family of CNS neurocytomas. To the best of our knowledge, there are only 21 cases of sellar/suprasellar neurocytoma reported in the English literature. The neuroimaging features of EVNs arising from the sellar/suprasellar region are indistinguishable from those seen in tumors of the pituitary gland. Histopathologically, the differential diagnosis of sellar/suprasellar EVNs includes pituitary adenoma/pituitary neuroendocrine tumors that can also invade the sinuses, paraganglioma, and olfactory neuroblastoma (4). The biological behavior of sellar/suprasellar EVNs is unclear because few cases have been reported. Here, we retrospectively analyzed the clinicopathological and molecular features of 4 cases of extraventricular neurocytoma arising in the sellar or suprasellar region and reviewed the related literature.

## Materials and Methods

Seven tumor samples from 4 patients with sellar/suprasellar EVN were retrieved from the archives of the Department of Pathology of the First Affiliated Hospital of Fujian Medical University and the Affiliated Hospital of Qingdao University between 2000 and 2020 with the approval of the research ethics board. Written informed consent was obtained from the individual(s) AND/OR minor(s)’ legal guardian/next of kin for the publication of any potentially identifiable images or data included in this article. Clinical data of the patients were obtained from their medical records.

All available immunohistochemical stains were reviewed and documented. If not performed at the time of pathological diagnosis, immunostaining including pituitary transcription factors and so on was performed for each tissue sample with the available formalin-fixed paraffin-embedded tissue blocks. Primary antibodies used for immunostaining are against the following proteins: pituitary-specific positive transcription factor 1 (Pit1), T-box transcription factor 19 (T-Pit), splicing factor 1(SF1), estrogen receptor alpha (ERα), GATA binding protein 2(GATA2), thyroid transcription factor 1 (TTF1, SPT24), synaptophysin(syn), chromogranin A(CgA), cytokeratin 8 (CK8), prolactin, growth hormone (GH), thyroid-stimulating hormone (TSH), adrenocorticotropic hormone (ACTH), follicle-stimulating hormone (FSH), luteinizing hormone(LH), S100, neuron-specific enolase (NSE), neuronal nuclei (NeuN), calretinin, glial fibrillary acidic protein (GFAP), oligodendrocyte transcription factor 2(Olig2), neurofilament protein (NF), Ki-67, GATA3, p53, E-cadherin, and vasopressin. Staining was performed on a BenchMark ULTRA system (Ventana Medical Systems, Tucson, AZ). A reticulin stain was also performed.

All 7 samples were tested by fluorescence *in situ* hybridization for bHLH transcription factor (MYCN), fibroblast growth factor receptor 1 (FGFR1), epidermal growth factor receptor (EGFR), 1p/19q and cyclin dependent kinase inhibitor 2A (CDKN2A) gene alterations and sequencing by Sanger sequencing for isocitrate dehydrogenase 1 (IDH1), IDH2, and BRAF V600E mutations. All probes were obtained from Gene Company Limited (Hong Kong).

## Results

### Clinical and Radiologic Findings

**Figure 1:**
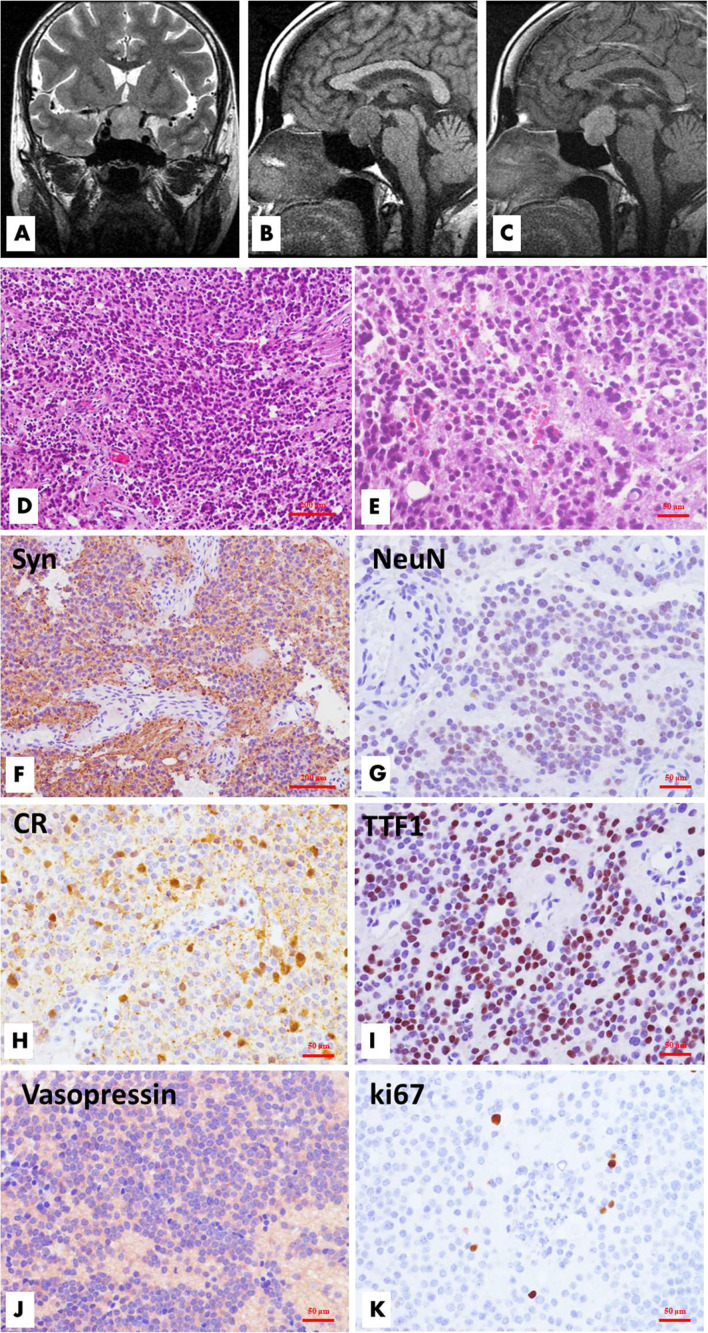
Radiological, histological and immunohistochemical features of sellar/suprasellar neurocytoma (Case 1). MR imaging demonstrated a well-circumscribed mass located in the sellar and suprasellar regions. The tumor was inhomogeneously hyperintense on coronal T2WI, and the left suprasellar sinus space was involved **(A)**, while the lesion was hypointense on sagittal T1WI, and the optic chiasma was displaced upwardly **(B)**. The lesion showed significant homogeneous enhancement on enhanced T1WI **(C)**. Microscopically, the tumor is comprised of solid nests or sheets of noncohesive monotonous small round cells with round to oval nuclei and fine chromatin. The poorly defined cytoplasm merges with the neuropil. Necrosis and mitotic figures are absent. **(D**, H&E, ×100; **E**, H&E, ×200**)** Immunohistochemical analysis revealed that the tumor cells had neuronal differentiation and were positive for synaptophysin **(F**, ×100**)**, NeuN **(G**, ×200**)**, and calretinin **(H**, ×200**)**. TTF1 **(I**, ×200**)** and vasopressin **(J**, ×200**)** had variable reactivities. The Ki-67 labeling index is approximately 1.5% **(K**, ×200**)**.

The study group included 3 women and 1 man whose age at the onset of symptoms ranged from 27 to 46 years with a median age of 34 years. Patients presented mainly with worsening visual disturbances and headaches for 5 months to 2 years. All patients had no abnormalities of adenohypophyseal hormones on the preoperative examination. CT scan demonstrated a well-circumscribed lesion isodense with the brain parenchyma. Focal calcification in the tumor was present in case 4. Brain magnetic resonance imaging (MRI) revealed a sellar solid mass, with focal cysts in cases 3 and 4 extending to the suprasellar region, causing enlargement of the sellar, infiltrating the cavernous sinus, and encasing the internal carotid artery. The masses were isointense or hypointense on T1-weighted imaging and heterogeneously hyperintense on T2-weighted imaging. Post-contrast T1 images showed moderate enhancement (**Figure 1**) (**Table 1**).

**Table 1:** Clinicopathological details of the present 4 cases of sellar/suprasellar neurocytoma.

case	Sex	Age	Initial symptoms	Serum vasopressin	Pituitary hormone	Location	Focal infiltrations	Preoperative impression	Type	Initial operation	Adjuvant radiotherapy	Recurrence (months)	second operation	Type	follow-up (months)
1	F	28	Visual disturbances; 2-year	normal	normal	S/S	yes	Pituitary adenoma	typical	STR	No	Yes (50)	STR	atypical	(live) 63
2	M	46	Visual impairment; 2-year	NA	NA	S/S	yes	Pituitary adenoma	typical	STR	No	Yes (118)	STR	typical	(live) 137
3	F	27	Blurred vision; 5-month	NA	normal	S/S	yes	Pituitary benign tumor	typical	GTR	No	No	No	No	(live) 65
4	F	40	Bitemporal hemianopsia; 10-month	NA	normal	S/S	yes	Pituitary adenoma	atypical	STR	No	Yes (11)	STR	atypical	(live) 23

NA, Not available; S/S, Sellar/Suprasellar; S, Sellar; PA, Pituitary adenoma; STR, Subtotal resection; GTR, Gross total resection.

### Histopathological Features

**Figure 2:**
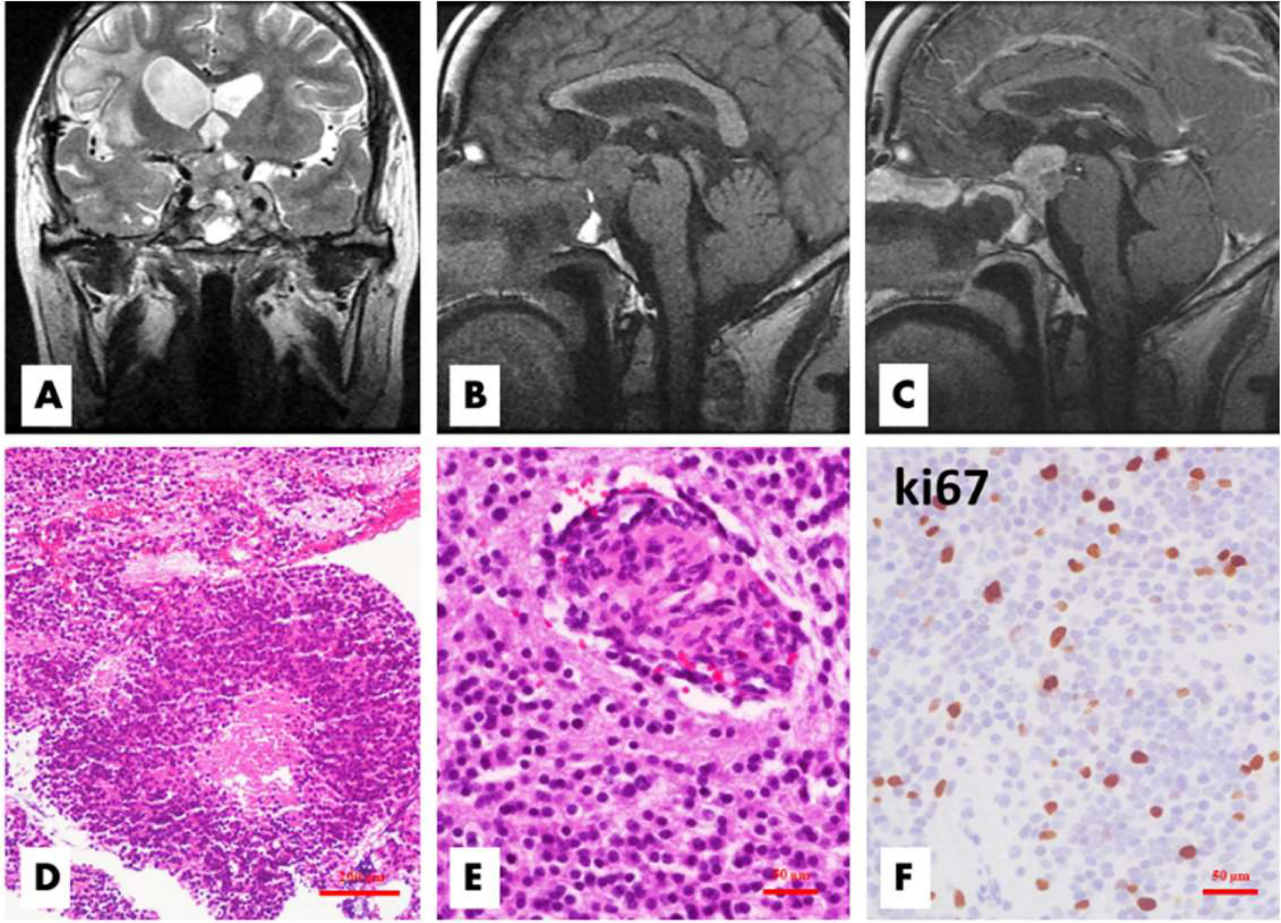
Patient 1 relapsed 50 months after the first surgery. The tumor located in the sellar and suprasellar regions demonstrated inhomogeneous hyperintensity on coronal T2WI, and the bilateral cavernous sinuses were involved **(A)**. On sagittal T1WI images, the lesion exhibited inhomogeneous hypointensity and irregular margins **(B)**, with significant inhomogeneous enhancement on enhanced T1WI **(C)**. Histologically, there were some atypical or anaplastic features, including focal necrosis **(D**, H&E, ×100**)** and microvascular proliferation **(E**, H&E,×200**)**, with a high Ki-67 index **(F**, ×200**)**.

Histopathological examination revealed a moderately hypercellular neoplasm comprised of sheets of small- to medium-sized cells with round-to-oval, monomorphic nuclei with dispersed chromatin and inconspicuous nucleoli (**Figure 1**). The nuclei were surrounded by scant finely granular eosinophilic cytoplasm or perinuclear halos. Focal areas of the tumor were less cellular and had anuclear areas with fine fibrillary neuropil. Rare mitotic figures were present. Necrosis and vascular proliferation were absent. There was focal fibrosis and calcification. The recurrent tumor tissues of case 1 (**Figure 2**) and case 4 showed atypical histologic features, including focal necrosis, microvascular proliferation, and active mitoses. In addition, the recurrence of case 1 was comprised of smaller-sized and partly markedly hypercellular tumor cells. Classical large gangliocytes were lacking in the tumor tissues of all 7 samples.

### Immunophenotypic Features and Molecular Genetics Results

Immunohistochemically, the tumor cells were positive for synaptophysin, chromogranin A, and calretinin and were focally positive for NeuN, TTF1, NF, CK8, vimentin, and S100 proteins. A few entrapped or reactive astrocytes expressed GFAP, but the tumor cells were negative. Other markers, including IDH1, BRAF VE1, Olig-2, EMA, E-cadherin and GATA3, were negative. All pituitary transcription factors, including Pit-1, T-pit, SF1, ER_α,_ and GATA2, and anterior pituitary hormones, such as GH, PRL, TSH, FSH, LH and ACTH, were negative. However, vasopressin expression was identified in all 7 samples. The Ki-67 labeling index of cases 1-3 was 1% to 2%, and it was 6% in case 4, while those of the recurrent cases 1, 2 and 4 were 5%, 2%, and 10%, respectively.

Sanger sequencing did not detect IDH1, IDH2 or *H3F3A* mutations in 5 of 7 FFPE samples (case 1 and its recurrence, recurrence of case 2 and case 4, and case 3), while loss of ATRX expression was absent by immunostaining. Fluorescence *in situ* hybridization (FISH) detection showed that tumor cells were intact on chromosomes 1p and 19q, CDKN2A nondeletion, and EGFR nonamplification. Rearrangement of fibroblast growth factor receptor 1 (*FGFR1*) was not found by *FGFR1 break*-apart *probe FISH.* The BRAF V600E mutation and TERT promoter mutations were negative by tetraprimer amplification refractory mutation system-polymerase chain reaction (ARMS-PCR), and the absence of O6-methylguanine-*DNA* methyltransferase (*MGMT*) gene promoter methylation was identified by pyrosequencing in the 3 cases of recurrence (**Table 2**).

**Table 2:** Immunohistochemical and molecular features of all 7 samples of 4 cases of sellar/suprasellar neurocytoma.

Case	Syn	NeuN	NF	TTF1	vasopressin	calretinin	MAP2	TFs	hormones	Ki67 (+,%)	IDH1/2 (Sanger Sequencing)	1p/19q (FISH)	EGFR (FISH)	TERT (Sanger sequencing)	CDKN2A (FISH)	FGFR1 (FISH)	MGMT (Pyro-sequencing)
1	++	f+	f+	S+	+	+	+	–	–	1.5	wildtype	intact	nonamp	wildtype	intact	nonrearrangement	NA
R1	++	f+	–	–	+	+	+	–	–	5	wildtype	intact	nonamp	wildtype	intact	nonrearrangement	unmethylated
2	++	+	f+	S+	+	+	+	–	–	1	NA	NA	NA	NA	NA	nonrearrangement	NA
R2	++	S+	f+	–	+	+	+	–	–	2	wildtype	intact	nonamp	wildtype	intact	nonrearrangement	unmethylated
3	++	+	f+	+	+	++	+	–	–	1.5	wildtype	intact	nonamp	wildtype	intact	nonrearrangement	NA
4	++	f+	f+	+	+	+	+	–	–	6	NA	NA	nonamp	NA	intact	nonrearrangement	NA
R4	++	f+	f+	S+	+	W+	+	–	–	10	wildtype	intact	nonamp	wildtype	intact	nonrearrangement	unmethylated

+, positive; ++, strongly positive; f, focally; s, scattered; w, weakly; -, negative; TFs, transcr factors of adenohypophysis; NA, not available; FISH, fluorescence in situ hybridization.

### Treatments and Prognosis

All 4 patients underwent surgical treatment. Case 2 was excised *via* a transsphenoidal procedure tumor resection, and transsphenoidal endoscopic approach resection of the mass was performed in the other three cases. The tumors in case 1, case 2 and case 4 were resected subtotally through the nasal sella. In case 4, only approximately 2/3 of the mass could be removed.

With a median follow-up of 74.5 months (range 23 to 137 months), all 4 patients survived. Case 1, case 2 and case 4 relapsed at 50, 118 and 11 months after initial surgery, and they underwent a transsphenoidal endoscopic approach resection for subtotal removal of the tumor again. Radiation therapy was performed after the second surgery (**Table 1**).

## Discussion

Sellar/suprasellar neurocytoma is an extraventricular neurocytoma arising from the hypothalamic-pituitary region. Only 21 cases of EVNs arising in the sellar/suprasellar region have ever been reported in the English literature. The clinical, radiological, and pathological features of the patients are shown in **Table 3**. The ages of the patients at first diagnosis ranged from 14 to 70 years, with an average of 44 years, and the female-to-male ratio was 1:1. The original complaints were reduced visual acuity with bitemporal or asymmetrical hemianopsia related to focal compression. Headache and dizziness would also occur. Radiological examination revealed that the sellar/suprasellar neurocytomas were generally well-defined, solid, homogeneous, and with limited or no peritumoral swelling. Small−scattered calcifications and cystic components occurred in a few cases. The vast majority of tumors, including the present 4 cases we reported, presented with invasion of the cavernous sinus. The CT scans revealed discreetly iso/hyperdense and heterogeneous enhancing appearances. In general, magnetic resonance imaging (MRI) showed that the EVNs were iso-signal to gray matter on T1- and T2-weighted images, with a variable heterogeneous enhancement pattern (6, 9, 23).

**Table 3:** Clinicopathological details of the 21 reported cases of sellar/suprasellar neurocytoma.

Case	Reference	Sex	Age	Initial symptoms	Serum vasopressin	Pituitary hormone	Location	Focal infiltrations	Preoperative impression	Type	Resection	Adjuvant radiotherapy	Recurrence
1	Maguire et al. (5)	F	55	Visual disturbances; 6 months	NA	normal	S/S	no	PA	typical	STR	NA	NA
2	Yang GF et al. (6)	F	46	Visual impairment; 1 year	NA	normal	S/S	yes	Meningioma	typical	STR	NA	NA
3	Chen H et al. (7)	M	52	Blurred vision; 6-month	NA	normal	S	no	NA	typical	GTR	no	NA
4	Wang Y et al. (8)	F	50	Decreasing vision; 2-month	NA	normal	S/S	yes	PA or craniopharyngioma	typical	STR	yes	no
5	Liu K et al. (9)	M	40	Visual impairment; NA	NA	normal	S/S	yes	PA	typical	NA	no	no
6	Wang Y et al. (10)	F	23	Bitemporal visual deficit and headache;4-month	NA	NA	S/S	yes	NA	typical	STR	yes	NA
7	Kawaji H et al. (11)	M	48	Visual impairment	NA	PRL↑	S/S	yes	PA	atypical	STR	yes	6 years
8	Xiong Z et al. (12)	NA	56	NA	NA	NA	S	NA	NA	typical	NA	NA	NA
9	Makis W et al. (13)	F	64	Bitemporal hemianopsia;30-year history of recurrent sellar masses	NA	NA	S/S	NA	Recurrent PA	atypical	NA	yes	30yrs ago(typical)
													4yrs ago(atypical)
10	Peng P et al. (14)	M	56	Bitemporal hemianopsia	NA	ACTH↓	S/S	yes	PA	typical	GTR	no	no
11	Chen S et al. (15)	F	50	Decreasing vision in left eye and diplopia; 2-month	NA	normal	S/S	yes	NA	typical	STR	yes	no
12	Chen S et al. (15)	M	62	Homonymous hemianopsia, temporal both eyes; 1 year	NA	PRL↓	S/S	no	NA	typical, ganglion	STR	yes	no
13	Cho et al. (16)	M	14	Bitemporal hemianopsia, decreased visual acuity; 0	NA	NA	S/S	yes	Hypothalamic glioma	typical	STR	yes	1 year
14	Wang et al. (16)	M	25	Worsening vision; 7-month	NA	normal	S/S	yes	PA or meningioma	typical	STR	yes	no
15	Tan CL et al. (17)	F	59	Visual disturbances; 2-year; with a history of osteoporosis and SIADH	NA	NA	S/S	yes	PA	typical	STR	yes	no
16	Nery B et al. (18)	M	27	Progressive bilateral vision loss; 4-year	NA	normal	S/S	yes	PA	typical	GTR	no	no
17	Tish S et al. (19)	M	70	Imbalance and dizziness	NA	normal	S/S	yes	NA	atypical, ganglion	biopsy	yes	no
18	Asa et al. (21)	F	39	5-month history of worsening visual field loss; idiopathic SIADH; 6-year	vasopressin excess with SIAD	normal	S/S	yes	PA	atypical	STR	no	NA
19	Asa et al. (21)	F	34	Galactorrhea, amenorrhea, hyponatremia; 18-month	vasopressin excess with SIAD	PRL↑	S/S	NA	PA	atypical	STR	yes	30yrs, death
20	Asa et al. (21); Zhang D et al. (22)	M	17	Progressive abdominal pain, nausea and emesis; 3-year	vasopressin excess with SIAD	low total testosterone	S	yes	NA	atypical	STR	no	no
21	Asa et al. (21)	F	40	Visual disturbance and headache	no	normal	S/S	yes	PA	atypical	STR	no	<1 year

NA, Not available; SIADH, Syndrome of inappropriate antidiuretic hormone secretion; S/S, Sellar/Suprasellar; S, Sellar; PA, Pituitary adenoma; STR, Subtotal resection; GTR, Gross total resection.

Histopathologically, EVNs arising in the sellar/suprasellar region have similar features of central neurocytoma and EVNs arising from other loci (4, 18, 19). These tumors are comprised of sheets of uniform round cells with “salt and pepper” chromatin and inconspicuous nucleoli. The tumor cells are usually embedded in a neuropil-like fibrillary background. Calcifications and ‘chicken wire’-like capillaries are also common. Ganglion cell differentiation is uncommon in central neurocytomas but is relatively frequent, up to approximately 50%, in extraventricular neurocytomas (24–26). EVN with ganglioid differentiation mainly arises from the frontal, temporal, and parietal lobes. There are only 2 cases of EVN with ganglioid differentiation occurring in the sellar/suprasellar region reported in the literature (20). No evidence of ganglioid differentiation was shown in any of the 7 sample tissues in this study. Necrosis and microvascular proliferation occur in a few cases with aggressive growth. In our cases, 2 recurrences showed anaplastic characteristics and progressed into atypical EVNs.

Immunohistochemistry confirmed that neurocytomas are positive for neuronal differentiation markers such as synaptophysin and chromogranin A and variable neurofilaments, NeuN and calretinin. Hypothalamic hormone vasopressin and focal TTF-1 expression are seen, and some researchers have suggested that these tumors may be derived from the basal hypothalamus, which could provide a useful diagnostic tool for differential diagnosis. They were all negative for pituitary transcription factors and hormones, GFAP, keratins, EMA, IDH1, BRAF VE1, Olig-2 and so on. Most CNs usually show a Ki-67 labeling index of less than 2%; however, a small portion of tumors that are termed atypical CNs are characterized by an increased Ki-67 labeling index of more than 2% or 3%. Atypical CN was recognized by the WHO in 2016, accounting for approximately 25% of all CNs (27). A strict definition of “atypical EVN” has not been clarified by the WHO, and the Ki-67 labeling index for the definition of atypia ranges from 2% to 5% among different studies (28, 29). Generally, atypical EVNs are characterized by increased mitotic activity or a higher Ki-67 labeling index and other atypical features, such as necrosis or microvascular proliferation (29, 30). Compared with atypical CNs, the proportion of atypical EVNs is approximately 30% higher than that of all EVNs. The atypical cases of sellar/suprasellar EVNs in the literature and this study had a similar rate, accounting for 32% (8/25).

To date, little is known about the molecular genetic features of extraventricular neurocytoma. A study from Slevers et al. revealed that the presence of FGFR1-TACC1 or FGFR3-TACC3 gene fusions in extraventricular neurocytoma located in the supratentorial brain is a frequent molecular event, especially the former, accounting for approximately 60% (3). However, these gene fusions have not been reported in EVN in the sellar or parasellar regions. In the present 4 patients, no FGFR1 gene breakage or rearrangement was found by FISH detection, suggesting that sellar/suprasellar neurocytomas may not have the same molecular biological characteristics as those originating from other parts. A microarray-based comparative genomic hybridization investigation revealed distinct profiles, with loss and gain of multiple chromosomal loci, which concluded that MYCN gene amplification, together with loss of BIN1 expression, were typical of central neurocytoma (31). EGFR amplification mutations have been reported in two cases of atypical EVNs (32, 33). In our cases, there was no amplification of MYCN or EGFR, and no alterations in IDH1, IDH2, BRAF V600E, 1p/19q, H3F3A or CDKN2A were found.

Several cases of sellar/suprasellar EVN reported in the literature have confirmed that the tumor cells are immunoreactive for vasopressin (5, 21). Through electron microscopic observation, Maguire et al. found that the perinuclear cytoplasm of the tumor cells contains neurosecretory granules, which are also packed in swollen neuritic processes that resemble Herring bodies (5). They interpreted the immunohistochemical and ultrastructural features of this tumor as suggestive of primary hypothalamic derivation. All samples in this study were positive for vasopressin, supporting the above view. Asa et al. reported that 3 patients with sellar/suprasellar EVN had a syndrome of inappropriate antidiuresis, which is associated with excess vasopressin production by tumors (21). However, serum vasopressin levels were not investigated preoperatively or postoperatively in our cases, which is a limitation of this study. Therefore, whether the serum level of vasopressin can hint at the diagnosis of this tumor is unclear, and more case studies are needed.

Most of the reported cases and the present cases were diagnosed as giant pituitary adenomas before surgery, and none of them were suspected to be EVNs based on preoperative imaging. A large pituitary adenoma suspected to involve the sellar/suprasellar region with invasion of the venous sinuses and nonfunctional clinical changes always has the possibility of being a neurocytoma. Histopathologically, pituitary endocrine tumors are the first differential diagnosis. In most cases, the histological characteristics of pituitary endocrine tumors as well as the pattern of expression of transcription factors and pituitary hormone markers can easily distinguish them. However, it is difficult to make a differential diagnosis from null cell adenoma, a very rare group of pituitary neuroendocrine tumors (PitNETs) that are negative for all adenohypophyseal hormones and transcription factors. The main points of differential diagnosis include the identification of neuropils and the expression of NeuN and TTF1 in sellar/suprasellar EVNs. In addition, we found that E-cadherin and P120 are also useful markers for differential diagnosis (unpublished), which are immune-positive in pituitary adenomas but immune-negative in neurocytomas. Other differential diagnoses include paraganglioma and olfactory neuroblastoma. Immunohistochemical markers are helpful. For example, paraganglioma generally expresses GATA3 and tyrosine hydroxylase, while neurocytoma does not (34).

Gross total resection is the preferred treatment for EVN (35, 36). EVN that occurs in the sellar/suprasellar region often invades the sphenoid sinus and cavernous sinus and usually compresses the optic nerve and envelops the internal carotid artery, so gross total resection is quite difficult, and thus subtotal resection is often performed. Adjuvant radiotherapy, either conventional or radiosurgery with gamma knife, can improve both local control and survival in patients who undergo subtotal resection (37, 38). The effect of chemotherapy on EVNs as an adjuvant management tool remains unclear. The overall prognosis of EVNs is good, although the recurrence rate is higher than that of CNs. In addition to incomplete resection affecting the prognosis, atypical or anaplastic features are associated with a higher recurrence rate and generally worse outcomes. In our cases, all 4 patients underwent surgical treatment radiotherapy or chemotherapy was not performed postoperatively, and 3 cases underwent subtotal resection have relapsed after initial surgery.

In summary, neurocytoma of the sellar/suprasellar region is an extremely rare tumor. The morphological features and immunophenotypes of a neurocytoma in the sellar/suprasellar region are similar to classic central neurocytoma. The tumor is often not completely resected, and adjuvant radiotherapy is feasible after surgery. Attention should be given to the differential diagnosis of pituitary endocrine tumors. The overall prognosis is good. Atypical histological features and subtotal resection may be related to tumor recurrence. Its biological behaviors and molecular genetics/epigenetics characteristics need to be studied further with a larger sample.

## Data Availability Statement

The original contributions presented in the study are included in the article/supplementary material. Further inquiries can be directed to the corresponding authors.

## Author Contributions

LFZ: Investigation, Data curation, Writing- Original draft preparation. WF: Investigation, Data curation, Writing- Original draft preparation. LMZ and YHY: Data curation, Visualization. FS: Methodology. YC: Methodology. CJ: Data curation. ZX: Data curation. CH: Data curation. SZ: Project administration. XY: Data curation, Supervision, Writing- Reviewing and Editing. XW: Funding acquisition, Supervision, Writing- Reviewing and Editing. All authors contributed to the article and approved the submitted version.

## Funding

This study was supported by Natural Science Foundation of Fujian Province (2018J01155), Fujian Medical University Startup Fund for scientific research (2020QH1053) and Fujian Provincial Health and Family Planning Training Project for Young and Middle-aged Key Talents (2019-ZQN-61).

## Conflict of Interest

The authors declare that the research was conducted in the absence of any commercial or financial relationships that could be construed as a potential conflict of interest.

## Publisher’s Note

All claims expressed in this article are solely those of the authors and do not necessarily represent those of their affiliated organizations, or those of the publisher, the editors and the reviewers. Any product that may be evaluated in this article, or claim that may be made by its manufacturer, is not guaranteed or endorsed by the publisher.
